# Public and Research Interest in Telemedicine From 2017 to 2022: Infodemiology Study of Google Trends Data and Bibliometric Analysis of Scientific Literature

**DOI:** 10.2196/50088

**Published:** 2024-05-16

**Authors:** Andrea Maugeri, Martina Barchitta, Guido Basile, Antonella Agodi

**Affiliations:** 1 Department of Medical and Surgical Sciences and Advanced Technologies “GF Ingrassia” University of Catania Catania Italy; 2 Department of General Surgery and Medical-Surgical Specialties University of Catania Catania Italy

**Keywords:** telemedicine, eHealth, digital medicine, COVID-19, Google Trends, bibliometric, Google, data spanning, COVID-19, accessibility, cost reduction, cost, noncommunicable disease, eHealth, mobile health, awareness, policy decision

## Abstract

**Background:**

Telemedicine offers a multitude of potential advantages, such as enhanced health care accessibility, cost reduction, and improved patient outcomes. The significance of telemedicine has been underscored by the COVID-19 pandemic, as it plays a crucial role in maintaining uninterrupted care while minimizing the risk of viral exposure. However, the adoption and implementation of telemedicine have been relatively sluggish in certain areas. Assessing the level of interest in telemedicine can provide valuable insights into areas that require enhancement.

**Objective:**

The aim of this study is to provide a comprehensive analysis of the level of public and research interest in telemedicine from 2017 to 2022 and also consider any potential impact of the COVID-19 pandemic.

**Methods:**

Google Trends data were retrieved using the search topics “telemedicine” or “e-health” to assess public interest, geographic distribution, and trends through a joinpoint regression analysis. Bibliographic data from Scopus were used to chart publications referencing the terms “telemedicine” or “eHealth” (in the title, abstract, and keywords) in terms of scientific production, key countries, and prominent keywords, as well as collaboration and co-occurrence networks.

**Results:**

Worldwide, telemedicine generated higher mean public interest (relative search volume=26.3%) compared to eHealth (relative search volume=17.6%). Interest in telemedicine remained stable until January 2020, experienced a sudden surge (monthly percent change=95.7%) peaking in April 2020, followed by a decline (monthly percent change=–22.7%) until August 2020, and then returned to stability. A similar trend was noted in the public interest regarding eHealth. Chile, Australia, Canada, and the United States had the greatest public interest in telemedicine. In these countries, moderate to strong correlations were evident between Google Trends and COVID-19 data (ie, new cases, new deaths, and hospitalized patients). Examining 19,539 original medical articles in the Scopus database unveiled a substantial rise in telemedicine-related publications, showing a total increase of 201.5% from 2017 to 2022 and an average annual growth rate of 24.7%. The most significant surge occurred between 2019 and 2020. Notably, the majority of the publications originated from a single country, with 20.8% involving international coauthorships. As the most productive country, the United States led a cluster that included Canada and Australia as well. European, Asian, and Latin American countries made up the remaining 3 clusters. The co-occurrence network categorized prevalent keywords into 2 clusters, the first cluster primarily focused on applying eHealth, mobile health (mHealth), or digital health to noncommunicable or chronic diseases; the second cluster was centered around the application of telemedicine and telehealth within the context of the COVID-19 pandemic.

**Conclusions:**

Our analysis of search and bibliographic data over time and across regions allows us to gauge the interest in this topic, offer evidence regarding potential applications, and pinpoint areas for additional research and awareness-raising initiatives.

## Introduction

The concept of telemedicine—defined as the use of technology to provide health care services remotely—has been around since the early 20th century, but it has gained significant attention globally only in recent years [1]. The development of technology such as videoconferencing, remote monitoring devices, and mobile health apps has made it possible to deliver health care services remotely, paving the way for the widespread adoption of telemedicine [2-4]. It is worth noting that the term telemedicine also covers health data analysis and the application of big data and artificial intelligence methods for epidemiological research and diagnosis support [3,5-7].

The use of technology allows patients to access care from anywhere, at any time and reduces the need for in-person visits, which can be particularly beneficial for individuals with mobility issues, those living in rural or remote areas, and individuals with chronic diseases [8,9]. The potential benefits of telemedicine are numerous, including increased access to health care services, reduced health care costs, and improved patient outcomes [8-11]. Telemedicine can also help to address workforce shortages in health care, particularly in rural and remote areas, by enabling health care providers to deliver care to patients in those regions without the need for travel [10,12].

The COVID-19 pandemic has underscored the significance of telemedicine in maintaining uninterrupted health care delivery while mitigating the risk of virus transmission [13-18]. As an illustration, in light of the imperative to reduce COVID-19 exposure among patients and health care providers, many elective surgical procedures were rescheduled, prompting surgeons to adopt telemedicine as an alternative for preoperative, follow-up, and urgent surgical care consultations [15,19]. The pandemic has therefore forced health care providers to adapt quickly to the new reality of delivering care remotely, leading to a significant increase in the adoption and use of telemedicine globally [3,20]. As demand grew exponentially during the pandemic period, even the telehealth market is expected to grow to US $218.5 billion by 2025 [3]. Nevertheless, even after the COVID-19 pandemic recedes, it is highly improbable that this mode of health care delivery will be disregarded.

Despite the potential benefits of telemedicine, its adoption and implementation have been slow in some regions. Barriers to adoption include regulatory challenges, technological limitations, and resistance from health care providers and patients. People living in poorer regions, women, the elderly, and those living in rural or remote areas are far less likely to be online than those in wealthier regions [1,11]. Of those connected, nearly 90% use mobile devices to access the internet, which might not be appropriate for delivering digital health services [3]. A digital divide also exists in terms of digital literacy or low skills, which is a concern for the poorest, elderly, and others with limited access to technology [3]. For these reasons, understanding the level of interest in telemedicine among the public and research community can help identify areas for improvement. Google Trends is a valuable tool for investigating the level of interest of the general public in a specific topic. As such, it has been used in previous research to analyze the public’s interest in various health and health care–related subjects [21-32]. The same applies to a topic such as telemedicine, for which there is little evidence. From a research standpoint, we have recently observed a significant increase in the number of publications related to telemedicine, indicating a growing interest in this field among researchers. An interest that needs to be mapped in terms of scientists and groups of researchers, countries that are contributing most to the research, and areas of applications.

Overall, this study aims to provide a comprehensive analysis of the level of public and research interest in telemedicine, also consider any potential impact of the COVID-19 pandemic. We therefore limit our analysis to the period between 2017 and 2022, a period of 6 years preceding and following the pandemic. We use 2 different approaches to analyze the level of interest in telemedicine. The first approach is an analysis of Google Trends data, which allows us to analyze the level of interest in telemedicine among the general public. The second approach is a bibliometric data analysis, which involves examining publications related to telemedicine over the past 6 years. Our analysis of search and bibliographic data over time and across regions helps us understand the level of interest in this topic, identify areas for further research and awareness-raising efforts, and inform policy decisions regarding telemedicine adoption and implementation.

## Methods

### Data Collection

#### Google Trends Data

Google Trends provides open access to time-series data related to Google searches for specific terms and topics [33]. According to the framework proposed by Mavragani and Ochoa [34], we retrieved Google Trends data separately by using the search topics “telemedicine” or “e-health,” encompassing all search categories. We queried Google Trends on March 9, 2023, and Google Trends data were obtained at the global level, as well as by country, for the period between January 1, 2017, and December 31, 2022. It is worth mentioning that search topics are a group of terms that share the same concept across languages, covering an array of variations, typos, and related searches [34,35]. This precludes the need to enter a set of individual keywords, while maintaining the consistency of search queries across all regions and timeframes [34,35]. Furthermore, using specific search topics without any search category restrictions proves beneficial for capturing the general interest of diverse populations [34,35]. We also obtained data for the “top related topics” that are most frequently searched with the topics under investigation (ie, telemedicine” or “e-health”).

In general, Google Trends data are provided as a normalized measure (ie, relative search volume [RSV]), obtained by dividing the search volume for a given term or topic by the total number of searches. This normalization process resulted in a percentage scale, with 100% corresponding to the peak in search volume in any given time frame and location. The value 0% does not necessarily indicate no searches, but rather a very low search volume for a given term or topic [34].

#### Bibliometric Data

Before describing the collection of bibliometric data, it is necessary to distinguish bibliometric analysis from reviews and systematic reviews of scientific literature. The first primarily uses a mechanistic method to track the global research trends in a certain field based on the outputs of scientific literature databases. Reviews and systematic reviews are instead characterized by methodical and replicable methodologies to find, select, and synthesize all available evidence on a particular topic or clinical question.

In our study, we searched the Scopus database for all articles mentioning the terms “telemedicine” or “eHealth” in the title, abstract, and keywords. We have chosen Scopus because it is recognized as the largest scientific literature database of peer-reviewed articles covering a wide range of subjects [36]. The literature search was conducted on 9th March 2023 and was limited to original articles published in English and in the subject area of medicine from 2017 to 2022. The query string used for the search was: TITLE-ABS-KEY (“telemedicine” OR “eHealth” ) AND PUBYEAR > 2016 AND PUBYEAR < 2023 AND ( LIMIT-TO ( DOCTYPE , “ar” ) ) AND ( LIMIT-TO ( LANGUAGE , “English” ) ) AND ( LIMIT-TO ( SUBJAREA , “MEDI” ) ). The Scopus search result was exported in the format of a CSV file with all data elements, including information on citation, bibliography, abstract, and keywords.

### Data Analysis

#### Google Trends Data Analysis

We first conducted a univariate analysis and compared the public interest on the topics of telemedicine and eHealth at the global level. Next, a joinpoint regression analysis was carried out to identify possible time points at which public interest trends changed. This analysis was conducted on log-transformed RSV, with 5000 permutations and assuming uncorrelated errors. The grid search method was chosen to determine where to locate joinpoints on the timescale [37]. Results were reported as the monthly percent change (MPC), calculated as the average percentage change per month between different joinpoints. Joinpoint regression analysis was performed using the Joinpoint Regression Program (version 4.3.1.0; Statistical Research and Applications Branch, National Cancer Institute) provided by the Surveillance, Epidemiology, and End Results Program (National Cancer Institute) on the website. We next mapped the public interest in telemedicine and eHealth by country, selecting the top 5 countries with the greatest interest. In these countries, Google Trends data were correlated with the number of new confirmed COVID-19 cases, deaths, hospitalizations, and patients in the intensive care unit per million residents. These data were obtained from the Our World in Data website [38]. Results were reported as the Spearman rank correlation coefficient (ρ). All statistical analyses were 2-tailed and performed with a significance level of 0.05.

#### Descriptive Analysis of Bibliometric Data

Descriptive analysis of bibliometric data was performed using Bibliometrix (K-Synth), an open-source R-tool for automating the stages of data analysis and data visualization [39]. After loading and converting bibliometric data in R (R Core Team), the main descriptive results were summarized as the number of documents, authors, sources, keywords, timespan, and average number of citations. Accordingly, tables and visualizations were obtained for the annual scientific production, top articles per number of citations, most productive authors, most productive countries, total citations per country, most relevant journals, and most relevant keywords.

#### Network Analysis of Bibliometric Data

Next, the VOSviewer software (version 1.6.16; Centre for Science and Technology Studies, Leiden University) was used to construct networks projecting authors’ and countries’ collaborations, as well as trending research topics through the analysis of keywords. This mapping method is generally used to estimate the association strength between different bibliometric items (ie, the nodes of the network), which may for example be publications, authors, or keywords [40]. The relation between 2 items is represented by a link (ie, the edges of the network), which may be a bibliographic coupling link between publications, a coauthorship link between authors, or a co-occurrence link between keywords. Each link has a strength, indicated by a positive number with higher values associated with stronger links [40]. The association strength may for example be indicated as the number of cited references 2 publications have in common for the bibliographic coupling link; as the number of publications 2 authors have coauthored for the coauthorship links; or as the number of publications in which 2 keywords occur together for the co-occurrence link [40]. Accordingly, each item receives different attributes, such as the weight and score attributes. Weight is a nonnegative numerical attribute indicating the importance of the item in the network. From a graphical perspective, items with higher weights are shown more prominently than those with lower weights [40]. Score attributes instead indicate additional numerical properties of the item, which can be only visualized in the overlay visualization of a map. There are also 2 standard weight attributes that can be used for descriptive purposes and computed for each item or the entire network, the links attribute and the total link strength attribute [40]. By creating the network map, items can be grouped into clusters, which correspond to linked items, labeled with colors and numbers.

We first performed network analyses of coauthorship at the author and country level, using the fractional method to reduce the influence of documents with many authors. With this approach, the strength of a coauthorship between 2 authors is determined considering the number of documents coauthored normalized for the total number of authors of each coauthored document. At the author level, we included authors who have published at least 20 articles on the topic, with no restrictions on the number of citations. At the country level, we included the first 50 countries with the greatest number of articles, with no restrictions on the number of citations. To identify research areas of greatest interest and their connections, we also applied a co-occurrence analysis of author keywords occurring more than 50 times.

## Results

### Public Interest Over Time

**Figure 1:**
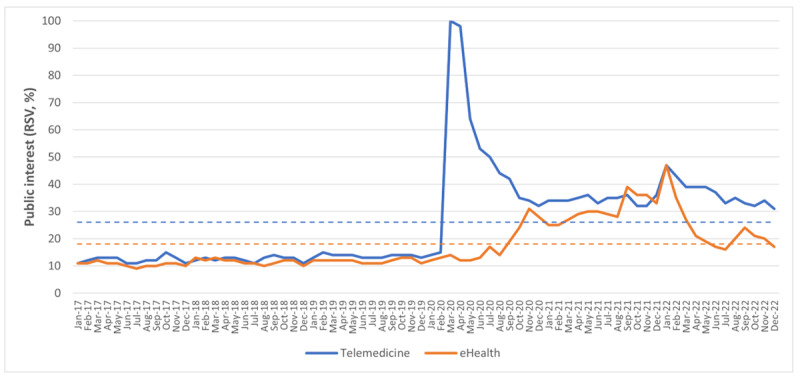
Public interest in telemedicine and eHealth from January 2017 to December 2020, was assessed through the Google Trends analysis. This graph shows the RSVs for the topics of telemedicine (blue line) and eHealth (orange line) over time. The dotted lines represent the average values. RSV: relative search volume.

Globally, the mean public interest in telemedicine and eHealth—expressed as RSV—was 26.3% (SD 18.1%) and 17.6% (SD 8.8%), respectively. The greater interest in telemedicine rather than in eHealth is evident from Figure 1, which shows RSVs from January 2017 to December 2022. According to the above figure, public interest in both topics was stable before the COVID-19 pandemic and then increased rapidly for telemedicine and more gradually for eHealth. Based on the joinpoint regression analysis (Figure S1 in Multimedia Appendix 1), public interest in telemedicine was stable until January 2020 (MPC=0.36%), which corresponded to the first joinpoint (37th month of the time series; 95% CI 36-38). Then, public interest suddenly increased (MPC=95.7%) to the highest peak reached in April 2020, which was the second joinpoint detected in the 40th month of the time series (95% CI 39-41). From that point on, public interest decreased (MPC=–22.7%) until August 2020 (ie, the third joinpoint on the 44th month; 95% CI 42-48) and then returned to being stable (MPC=–0.14%). Similarly, public interest in eHealth was stable until January 2020 (MPC=0.34%), which again was the first joinpoint of the time series (37th month; 95% CI 32-43). From that point on, public interest gradually increased (MPC=5.5%) to the highest peak reached in January 2022, which corresponded to the second joinpoint detected in the 61st month of the time series (95% CI 46-63). Then, public interest rapidly decreased (MPC=–20.1%) until May 2022 (ie, the third joinpoint on the 65th month; 95% CI 58-67) and then returned to being almost stable (MPC=1.8%).

### Geographic Distribution of Public Interest and Correlations With COVID-19 Data

**Figure 2:**
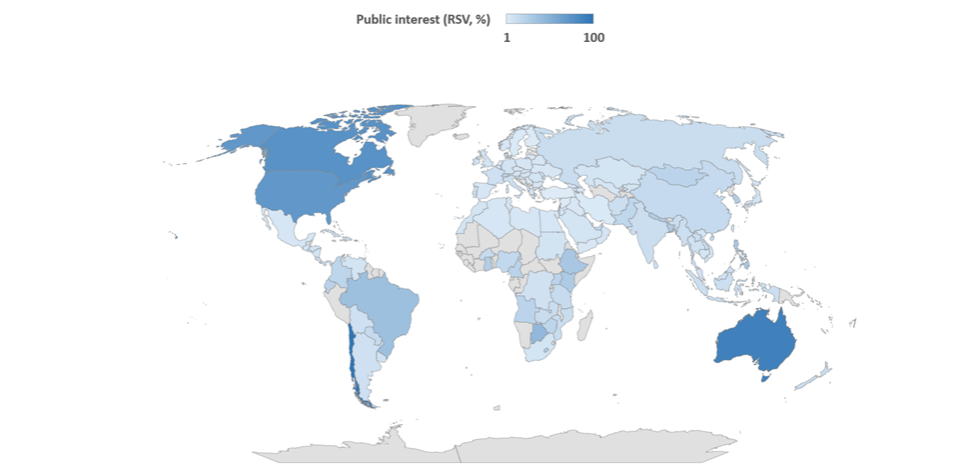
Geographic distribution of the public interest in telemedicine from January 2017 to December 2022, was assessed through the Google Trends analysis. This choropleth map illustrates the RSVs for the topic of telemedicine at the country level. RSV: relative search volume.

The map in Figure 2 shows the geographic distribution of public interest in telemedicine from 2017 to 2022. The top 5 countries were Chile, Australia, Canada, the United States, and Puerto Rico (Figure S2 in Multimedia Appendix 1). Public interest in eHealth was less widespread (Figure S3 in Multimedia Appendix 1), therefore we have not considered this topic for further analyses. For those countries included in the top 5, we evaluated correlations between the RSV for telemedicine and COVID-19 data per million residents. Public interest in Chile was strongly correlated with the number of new cases (ρ=0.718; *P*<.001), and weakly with the number of new deaths (ρ=0.321; *P*=.006). In Australia, public interest moderately correlated with the number of new cases (ρ=0.537; *P*<.001), new deaths (ρ=0.505; *P*<.001), hospitalized cases (ρ=0.559; *P*<.001), and patients in the intensive care unit (ρ=0.483; *P*<.001). Public interest in Canada was weakly correlated with the number of new cases (ρ=0.265; *P*=.001) and deaths (ρ=0.333; *P*<.001). In the United States, public interest was weakly correlated with the number of new deaths (ρ=0.295; *P*<.001). No correlations were evident for the public interest in Puerto Rico.

### Common Topics Related to Telemedicine

Despite the considerable impact of the pandemic on the public interest in telemedicine, there were few COVID-19-related topics among those that were commonly searched with telemedicine (Figure S4 in Multimedia Appendix 1). In particular, Google users interested in telemedicine mainly searched for general topics including health, physician, health care, medicine, and therapy. Moreover, there were some common topics related to specific telemedicine providers (eg, “Telehealth Ontario,” “Medicare,” “Santa Catarina,” “Cigna,” and “CVS Pharmacy”).

### Description of Bibliometric Data

We identified 19,539 original medical articles published in English and indexed in the Scopus database from 2017 to 2022. These articles were published by 2824 different sources (eg, journals and books), with a document average age of 2.8 years. Notably, the first 2 sources (JMIR and Telemedicine and eHealth) published a number of articles that were nearly 40% of the sum of the first 10 sources (Table S1 in Multimedia Appendix 1). The overall average number of citations per document was 11.2, while the average number of citations per document and per year was 2.6. The top 10 articles per citation are reported in Table S2 in Multimedia Appendix 1, their total citations ranged from 506 to 999, while their total citations per year ranged from 121 to 221. A total of 553,913 references were found in the bibliometric analysis of all the articles. The top 10 cited references are reported in Table S3 in Multimedia Appendix 1, with total citations ranging from 70 to 161.

### Research Interest Over Time

**Figure 3:**
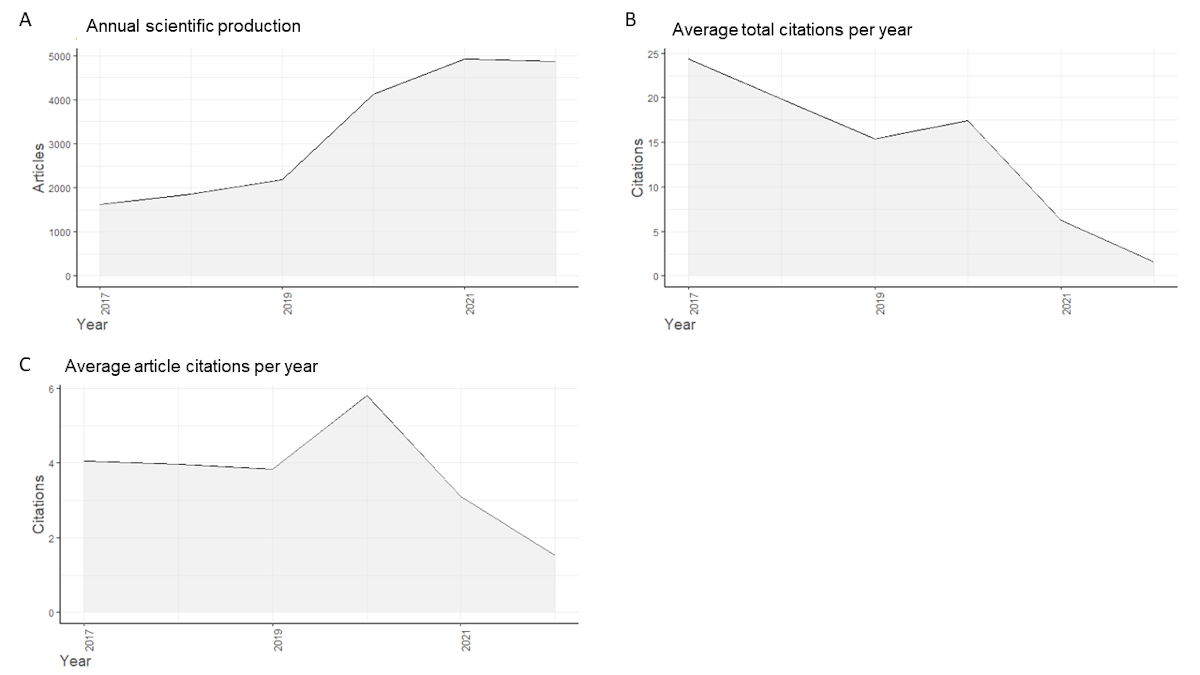
Research interest in telemedicine from 2017 to 2022, assessed through the analysis of bibliometric data. (A) Number of publications included in the bibliometric analysis from 2017 to 2022. (B) Average total citations per year from 2017 to 2022. (C) Average article citations per year from 2017 to 2022.

Our analysis of the bibliometric data also revealed a significant increase in the number of publications related to telemedicine over the past 6 years (Figure 3A). The number of publications per year increased from 1615 in 2017 to 4870 in 2022, showing an overall increase of 201.5% and an annual average growth rate of 24.7%. The greatest increase has been reported between 2019 and 2020, with an annual growth rate of 89.0%. Regarding citations, the average total citations per year decreased from 2017 to 2022 (Figure 3B), probably as a consequence of different document ages. By contrast, the average article citations per year were stable from 2017 to 2019, followed by a peak in 2020, and then decreased until 2022 (Figure 3C).

### Most Productive Authors and Coauthorship Network

**Figure 4:**
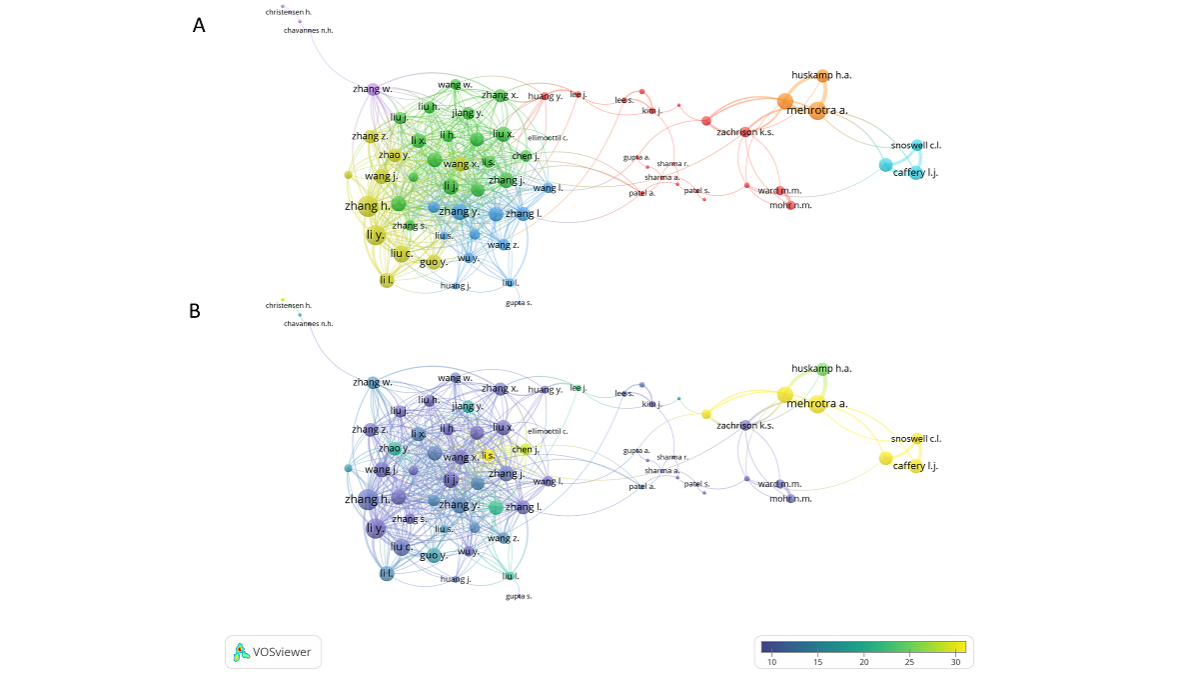
Coauthorship network of the most productive authors. The graphs show coauthorships between 71 authors with at least 20 publications on telemedicine, weighted by number of links. (A) Network visualization representing 7 clusters of collaborations. (B) Overlay visualization weighted by the number of links and scored by the average number of citations. The figures were prepared using VOSviewer.

Among the included articles, 93,394 authors were appearing 136,956 times overall. In particular, there were 690 single-authored documents written by 642 independent authors. Accordingly, the number of documents per author was 0.2, while the number of coauthors per document was 7.0. Table S4 in Multimedia Appendix 1 shows the list of the 10 most productive authors in terms of total and fractionalized articles. Their contribution to the field ranged from 48 to 80 articles (7.2 to 10.0 fractionalized articles). In Figure 4 it is shown that 71 of the authors who published at least 20 documents (n=80) were well connected. There were 488 links between these authors—which were grouped into 7 clusters—with a total link strength of 988.

### Most Contributing Countries and International Collaboration Network

The majority of publications came from a single country, while 20.8% featured international coauthorships. Table 1 shows the top 10 corresponding author’s countries per document, also considering differences between single-country and multiple-country publications. One thing to note was that the United States alone published almost the same number of articles as the other 9 (6610 vs 6653), representing 33.8% of all articles analyzed. Countries with the highest proportion of multiple-country publications were Germany (31.5%), the United Kingdom (27.9%), and the Netherlands (26.8%); those with the lowest proportion were the United States (11.4%), India (20.3%), and Italy (22.5%). Similar results were evident in terms of citations, with average citations per document ranging from 8.9 for Spain to 15.2 for India (Table S5 in Multimedia Appendix 1).

**Table 1:** Top 10 corresponding authors’ countries per document from 2017 to 2022.

Countries	Single country publications, n	Multiple country publications, n
United States	5858	752
Australia	792	281
United Kingdom	745	288
Canada	629	219
China	532	180
Germany	475	218
Netherlands	489	179
Italy	504	146
Spain	405	123
India	357	91

**Figure 5:**
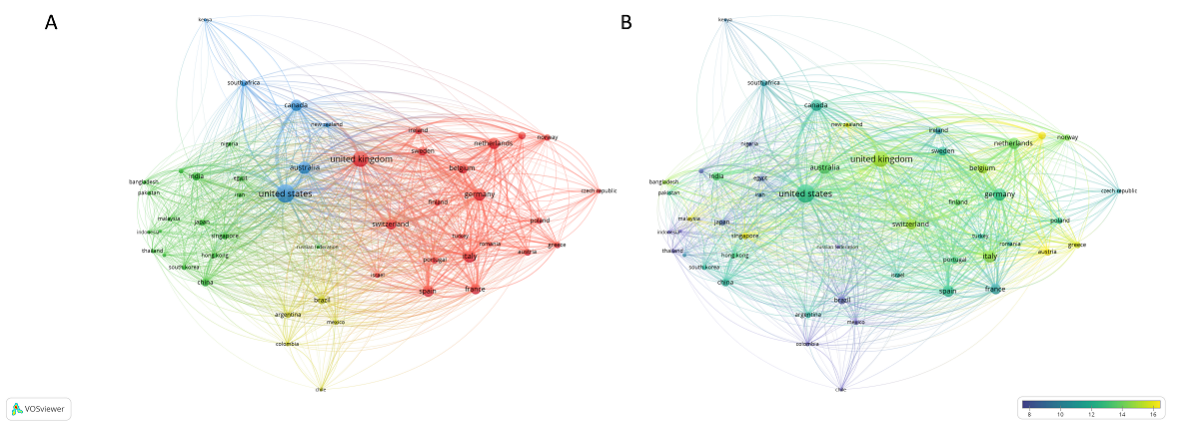
Collaboration network of the most contributing countries. The graphs show collaborations between the top 50 most contributing countries, weighted by the number of documents. (A) Network visualization representing 4 clusters of countries. (B) Overlay visualization weighted by the number of links and scored by the average number of citations. The figures were prepared using VOSviewer.

The coauthorship network based on the top 50 most contributing countries is illustrated in Figure 5. Overall, there were 1051 links with a total link strength of 14,751. Accordingly, countries were divided into 4 clusters. Cluster 1 consisted exclusively of European countries (n=21) with the United Kingdom, Germany, and Italy being the most interconnected; cluster 2 consisted of Asian countries (n=18) led by India and China; cluster 3 consisted of 6 countries, with the United States, Canada, and Australia being the most interconnected; and cluster 4 consisted of 5 countries of Latin America (ie, Brazil, Argentina, Chile, Colombia, and Mexico).

### Most Relevant Keywords and Co-Occurrence Network

Overall, 22,798 author’s keywords were found among the included studies. Based on the analysis of the most common keywords, COVID-19-related terms appear in the top 10 (Table S6 in Multimedia Appendix 1). In particular, this ranking put “COVID-19” and “pandemic,” respectively, in the 2nd and 8th place. In addition to common terms related to telemedicine (eg, telehealth, eHealth, and mobile health [mHealth]), the analysis also revealed mental health as an area of relevant interest.

**Figure 6:**
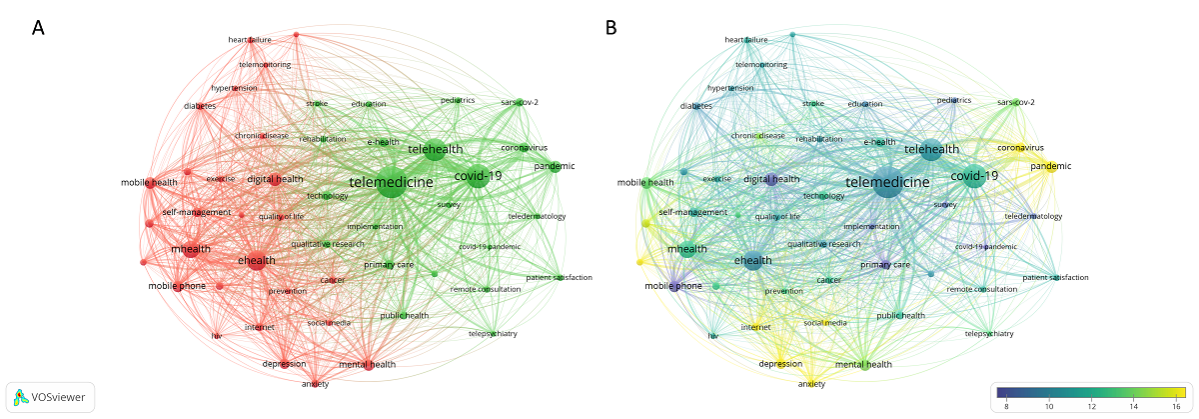
Co-occurrence network of the most common keywords. The graphs show the co-occurrence of the top 50 most common keywords, weighted by the number of links. (A) Network visualization representing 2 clusters of common keywords. (B) Overlay visualization weighted by the number of links and scored by the average number of citations. The figures were prepared using VOSviewer.

The co-occurrence network based on the top 50 most common keywords is illustrated in Figure 6. Overall, there were 1030 links with a total link strength of 21,275. Accordingly, keywords were divided into 2 clusters. Cluster 1 seemed mostly related to the application of eHealth, mHealth, or digital health to noncommunicable or chronic diseases (eg, diabetes, hypertension, depression, and anxiety) and cluster 2 was instead related to the application of telemedicine and telehealth in the context of the COVID-19 pandemic.

## Discussion

### Principal Results and Comparison With Prior Work

Our research highlights the increasing interest in telemedicine among the general public and researchers, particularly in response to the COVID-19 pandemic. Even prior to the outbreak, telemedicine had already demonstrated significant potential across various health care sectors. It offered a practical solution for delivering remote consultations, conducting preoperative evaluations, and facilitating postoperative follow-ups, particularly for patients residing in remote or underserved areas [9,19]. Although telemedicine comes with numerous advantages, it also presents certain drawbacks. For example, it may prove unsuitable for emergency situations requiring immediate hands-on medical attention, necessitating traditional, in-person emergency care [41]. Additionally, specific diagnostic procedures, like imaging or laboratory tests, may demand specialized equipment unavailable in a patient’s home, potentially impacting diagnostic accuracy [42]. Furthermore, not all individuals have access to the requisite technology for telemedicine consultations, such as a reliable internet connection, a suitable device, or the technical skills for virtual appointments [43]. This discrepancy in access can result in health care disparities. Finally, telemedicine involves transmitting sensitive health information over digital networks, posing challenges in ensuring the privacy and security of patient data and carrying a risk of data breaches or unauthorized access [43].

Despite these advantages and disadvantages, the pandemic served as a catalyst for the widespread adoption and acceptance of telemedicine [14,15,18]. Notably, telemedicine has garnered increased attention, especially in the aftermath of the COVID-19 pandemic, where its significance became evident due to the implementation of social distancing measures. However, limited studies have explored the population-level interest in telemedicine, as measured by tools such as Google Trends. Some of these studies are summarized in Table S7 in Multimedia Appendix 1 [44-51]. To the best of our knowledge, our study provides the most recent and comprehensive analysis in this field, shedding light on the level of interest among both the general public and researchers. An example of the use of infodemiological methods to explore global interest in telemedicine during the COVID-19 pandemic can be seen in the study conducted by Leochico et al [51] in 2020. Their study revealed a significant surge in online searches for telemedicine and related terms after the outbreak of the pandemic [51]. Extending the analysis period until the end of 2022, our study showed that while the level of public interest in telemedicine experienced a significant increase from January to April 2020, it later declined until August 2020 and eventually stabilized. Nevertheless, public interest has remained slightly higher compared to the pre-pandemic period. This finding is consistent with the results of a Google Trends analysis conducted by Wong et al [47] on the 50 countries most affected by the COVID-19 pandemic until July 2020. According to our findings, Chile, Australia, Canada, and the United States demonstrated the highest levels of public interest in telemedicine. In these countries, we observed moderate to strong correlations between Google Trends and COVID-19 data, including new cases and deaths, as well as hospitalizations. This finding is consistent with the results reported by Arshad Ali et al [45], who found a significant global correlation between the increase in COVID-19 cases and deaths and the interest in telemedicine.

With regard to research interest, there are examples of bibliometric analyses that existed even before the COVID-19 pandemic. For instance, Armfield et al [52] analyzed nearly 18,000 publication records, published between 2009 and 2013, to investigate the themes in telemedicine and telehealth literature. They found that the majority of studies focused on the clinical effectiveness of telemedicine. Other research questions include the adoption and implementation of telemedicine and eHealth technologies in health care systems [52]. Edirippulige et al [53] conducted a bibliometric analysis of telemedicine-related literature published until 2018 in highly ranked clinical journals and revealed that the acceptance of telemedicine research by these journals indicated a maturing of the telemedicine field. However, the pandemic has led to a surge of research interest in telemedicine and related fields. Our bibliometric analysis, in fact, revealed a considerable increase in the number of publications, particularly from 2020 onwards. Most of these publications came from a single country, with only 1 in 5 featuring international collaborations. Despite the importance of fostering collaborations among various stakeholders, including academics, health administrators, practitioners, policymakers, and communities, which involve reciprocal knowledge translation, such partnerships are often lacking [54]. The United States led the way as the most productive country, with Canada and Australia following in a cluster. Meanwhile, European, Asian, and Latin American countries comprised the other 3 clusters. Previous studies already demonstrated the predominant role of the United States, with Lan et al [55] providing a general overview, and Kumar et al [56] analyzing the trends in orthopedics and trauma-related telemedicine during the COVID-19 pandemic. Another aspect of our analysis focused on whether the different terms, such as telemedicine, telehealth, eHealth, mHealth, and so forth, can be used interchangeably or if each of them refers to a specific area of research. Fatehi and Wootton [57] conducted a bibliometric analysis in 2012 to examine the trends in the use of terms such as telemedicine, telehealth, and eHealth. They discovered that these terms were frequently used interchangeably, with a growing prevalence of the term “eHealth” in more recent years. On the contrary, our analysis revealed a specific focus on the use of eHealth, mHealth, or digital health for noncommunicable and chronic diseases, while telemedicine and telehealth were predominantly used in the context of the COVID-19 pandemic. The analysis by Lan et al [55], limited to the application of telemedicine to COVID-19, showed similar results. In particular, telemedicine was mainly used to provide mental health services, health care services delivery, and to control cross-infection. In contrast, the term “mobile apps” was closely associated with chronic illness entities such as diabetes, heart failure, and asthma, as well as health service entities such as patient education and self-care [55].

### Limitations

While Google Trends provides a valuable tool for analyzing public interest, there are several limitations to this approach. First, Google Trends only provides information on internet searches and does not account for offline discussions, media coverage, or other forms of engagement with the topic. This means that our results may not be representative of the entire population or capture the full extent of public interest. Second, the data provided by Google Trends are aggregated and anonymous, making it difficult to determine the specific demographics, motivations, or intentions behind the searches. This can limit the ability to draw meaningful conclusions or make accurate predictions about public behavior or attitudes toward telemedicine. Third, Google Trends data may be subject to various biases, such as the effect of media coverage or search engine optimization strategies. Additionally, the results may be influenced by factors such as seasonality, news events, or changes in search algorithms, making it challenging to compare trends over time or across different regions. Some of these limitations also apply to bibliometric analysis. First, bibliometric data may not reflect the complete picture of research interest as not all research is published and indexed in databases. Some research may be unpublished or published in non-indexed sources. Second, bibliometric analysis may not capture changes in research interest in real time. It can take some time for research to be published, indexed, and reflected in bibliometric data, meaning that the data may not reflect the most current state of research interest. Third, bibliometric analysis may not capture the full range of research interest as it is limited to the keywords used in publications. For instance, to offer a more comprehensive overview, one might consider examining interest in other trending subjects, like generative artificial intelligence and large language models. Finally, bibliometric analysis does not provide insights into the reasons behind the trends observed. It is limited to providing quantitative data on the number and frequency of publications and citations, and cannot provide qualitative insights into the motivations or drivers behind the research interest. For all these reasons, while the analysis of Google Trends and bibliometric data can provide a useful starting point for understanding public and research interest in telemedicine, it is essential to supplement this analysis with other sources of data and to interpret the results with caution.

### Conclusions

Our study offers a comprehensive picture of the evolving landscape of telemedicine and its growing importance in health care delivery. By analyzing search and bibliographic data across regions and over time, our study provides valuable insights into the level of interest in telemedicine. This information serves to pinpoint potential application fields, identify gaps in this research, and emphasize areas that warrant additional attention and efforts in raising awareness.

## Appendix

Multimedia Appendix 1 Additional outputs from the examination of Google Trends and bibliometric data.

## Abbreviations

mHealth: mobile health
MPC: monthly percent change
RSV: relative search volume

## Data Availability

The data sets analyzed during this study are available from the corresponding author on reasonable request.
