# Supplementary material for: Public and Research Interest in Telemedicine From 2017 to 2022: Infodemiology Study of Google Trends Data and Bibliometric Analysis of Scientific Literature
Source: J Med Internet Res. 2024 May 16;26:e50088. doi: 10.2196/50088 (PMC11140276; doi:10.2196/50088)
Supplement: Multimedia Appendix 1 [file jmir_v26i1e50088_app1.docx]

**Figure S1.** Joinpoint regression analysis of public interest in telemedicine.

**
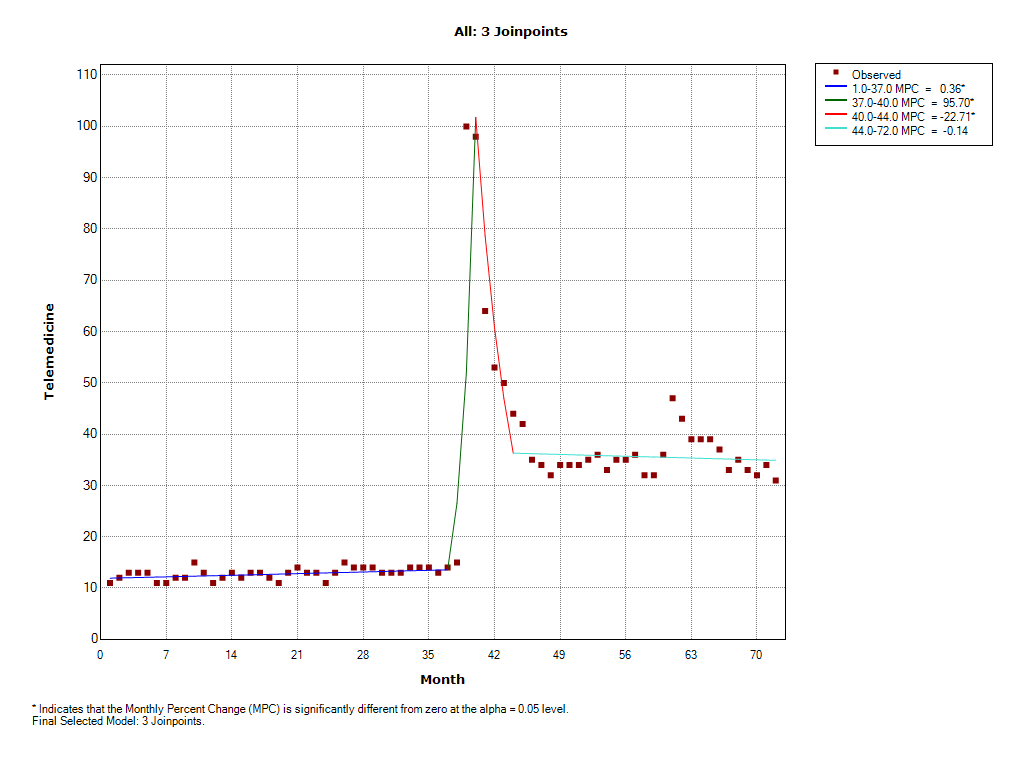
Figure S2.** Countries with the highest public interest in telemedicine from January 2017 to December 2022.

**
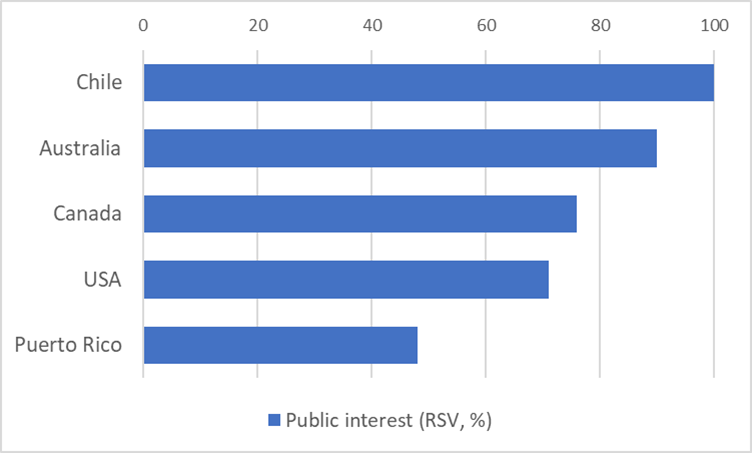
**

**Figure S3.** Geographic distribution of the public interest in e-health from January 2017 to December 2022.

**
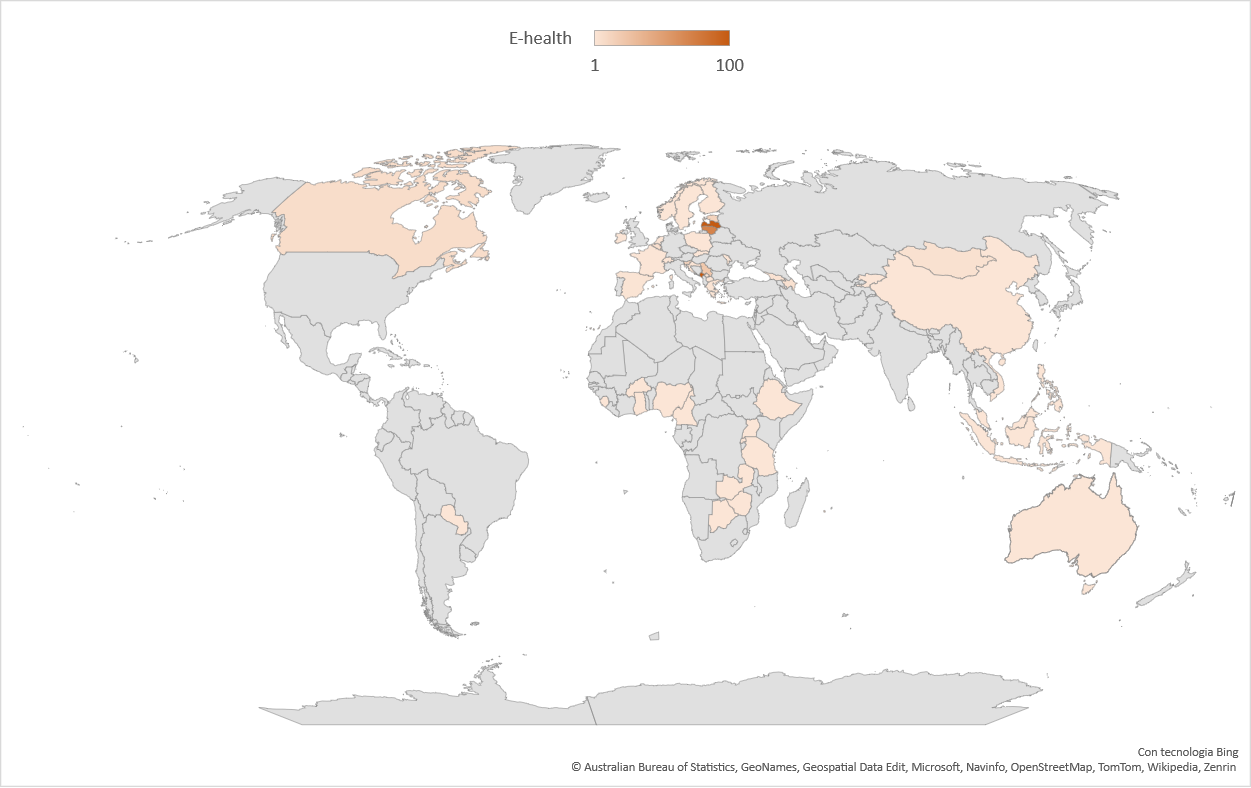
**

**Figure S4.** Commonly searched terms with telemedicine.


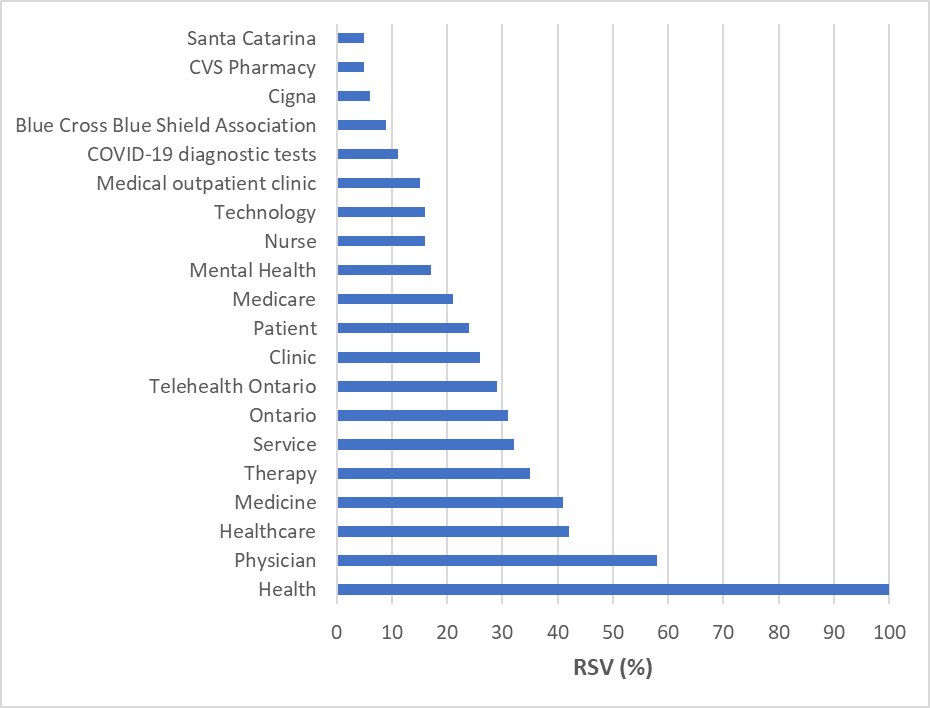


**Table S1.** Most relevant sources.

| Ranking | Sources | Number of articles |
| --- | --- | --- |
| 1 | JOURNAL OF MEDICAL INTERNET RESEARCH | 973 |
| 2 | TELEMEDICINE AND E-HEALTH | 817 |
| 3 | JMIR MHEALTH AND UHEALTH | 483 |
| 4 | JOURNAL OF TELEMEDICINE AND TELECARE | 464 |
| 5 | INTERNATIONAL JOURNAL OF ENVIRONMENTAL RESEARCH AND PUBLIC HEALTH | 456 |
| 6 | BMJ OPEN | 445 |
| 7 | JMIR RESEARCH PROTOCOLS | 247 |
| 8 | JMIR FORMATIVE RESEARCH | 242 |
| 9 | BMC HEALTH SERVICES RESEARCH | 185 |
| 10 | INTERNATIONAL JOURNAL OF MEDICAL INFORMATICS | 184 |

**Table S2.** Top manuscripts per citations.

| Ranking | First author | Year | Sources | DOI | Total citations | Total citations per year | Normalized total citations |
| --- | --- | --- | --- | --- | --- | --- | --- |
| 1 | Rajkomar A | 2018 | NPJ DIGIT MED | 10.1038/s41746-018-0029-1 | 999 | 166 | 50.4 |
| 2 | Smith AC | 2020 | J TELEMED TELECARE | 10.1177/1357633X20916567 | 884 | 221 | 50.8 |
| 3 | Dubey S | 2020 | DIABETES METAB SYNDR CLIN RES REV | 10.1016/j.dsx.2020.05.035 | 858 | 214 | 49.3 |
| 4 | Nahum-Shani I | 2018 | ANN BEHAV MED | 10.1007/s12160-016-9830-8 | 724 | 121 | 36.5 |
| 5 | Dashraath P | 2020 | AM J OBSTET GYNECOL | 10.1016/j.ajog.2020.03.021 | 687 | 172 | 39.5 |
| 6 | Perez MV | 2019 | NEW ENGL J MED | 10.1056/NEJMoa1901183 | 669 | 134 | 43.5 |
| 7 | Mann DM | 2020 | J AM MED INFORMATICS ASSOC | 10.1093/jamia/ocaa072 | 638 | 160 | 36.7 |
| 8 | Spinelli A | 2020 | BR J SURG | 10.1002/bjs.11627 | 540 | 135 | 31 |
| 9 | Breslin N | 2020 | AMERICAN J OBSTET GYNECOL MFM | 10.1016/j.ajogmf.2020.100118 | 517 | 129 | 29.7 |
| 10 | Chick RC | 2020 | J SURG EDUC | 10.1016/j.jsurg.2020.03.018 | 506 | 126 | 29.1 |

**Table S3.** Top ten cited references.

| Ranking | Reference | Number of citations |
| --- | --- | --- |
| 1 | Cohen, J. (1988). Statistical Power Analysis for the Behavioral Sciences (2nd ed.). Routledge. https://doi.org/10.4324/9780203771587 | 161 |
| 2 | Braun, V., & Clarke, V. (2006). Using thematic analysis in psychology. Qualitative Research in Psychology, 3(2), 77–101. https://doi.org/10.1191/1478088706qp063oa | 133 |
| 3 | Eysenbach G. The law of attrition. J Med Internet Res. 2005 Mar 31;7(1):e11. doi: 10.2196/jmir.7.1.e11. PMID: 15829473; PMCID: PMC1550631. | 132 |
| 4 | Hollander JE, Carr BG. Virtually Perfect? Telemedicine for Covid-19. N Engl J Med. 2020 Apr 30;382(18):1679-1681. doi: 10.1056/NEJMp2003539. Epub 2020 Mar 11. PMID: 32160451. | 110 |
| 5 | Braun, V., & Clarke, V. (2006). Using thematic analysis in psychology. Qualitative Research in Psychology, 3(2), 77–101. https://doi.org/10.1191/1478088706qp063oa | 108 |
| 6 | Eysenbach G. What is e-health? J Med Internet Res. 2001 Apr-Jun;3(2):E20. doi: 10.2196/jmir.3.2.e20. PMID: 11720962; PMCID: PMC1761894. | 86 |
| 7 | Bandura, A., & National Inst of Mental Health. (1986). Social foundations of thought and action: A social cognitive theory. Prentice-Hall, Inc. | 79 |
| 8 | Tong A, Sainsbury P, Craig J. Consolidated criteria for reporting qualitative research (COREQ): a 32-item checklist for interviews and focus groups. Int J Qual Health Care. 2007 Dec;19(6):349-57. doi: 10.1093/intqhc/mzm042. Epub 2007 Sep 14. PMID: 17872937. | 76 |
| 9 | American Psychiatric Association, DSM-5 Task Force. (2013). Diagnostic and statistical manual of mental disorders: DSM-5™ (5th ed.). American Psychiatric Publishing, Inc.. https://doi.org/10.1176/appi.books.9780890425596 | 72 |
| 10 | Norman CD, Skinner HA. eHEALS: The eHealth Literacy Scale. J Med Internet Res. 2006 Nov 14;8(4):e27. doi: 10.2196/jmir.8.4.e27. PMID: 17213046; PMCID: PMC1794004. | 70 |

**Table S4.** Most productive authors.

| Ranking | Authors | Articles | Authors | Articles Fractionalized |
| --- | --- | --- | --- | --- |
| 1 | Li Y | 80 | Li J | 10.03 |
| 2 | Li J | 63 | Mehrotra A | 9.9 |
| 3 | Wang Y | 60 | Wang Y | 9.41 |
| 4 | Liu Y | 55 | Li Y | 9.07 |
| 5 | Zhang Y | 53 | Mars M | 8.63 |
| 6 | Mehrotra A | 50 | Chen J | 8.51 |
| 7 | Wang J | 49 | Smith AC | 8.03 |
| 8 | Zhang J | 49 | Liu Y | 7.68 |
| 9 | Chen J | 48 | Price S | 7.36 |
| 10 | Li X | 48 | Kim J | 7.23 |

**Table S5.** Total citations per country.

| Ranking | Country | Total citations | Average article citations |
| --- | --- | --- | --- |
| 1 | USA | 81631 | 12.35 |
| 2 | Australia | 13698 | 12.77 |
| 3 | United Kingdom | 12986 | 12.57 |
| 4 | Canada | 8674 | 10.23 |
| 5 | Italy | 8669 | 13.34 |
| 6 | Netherlands | 7910 | 11.84 |
| 7 | Germany | 7681 | 11.08 |
| 8 | China | 7633 | 10.72 |
| 9 | India | 6821 | 15.23 |
| 10 | Spain | 4684 | 8.87 |

**Table S6.** Most relevant keywords.

| Ranking | Author keywords | Articles | Keywords-plus | Articles |
| --- | --- | --- | --- | --- |
| 1 | TELEMEDICINE | 6427 | TELEMEDICINE | 26619 |
| 2 | COVID-19 | 3149 | HUMAN | 17181 |
| 3 | TELEHEALTH | 2418 | FEMALE | 16175 |
| 4 | EHEALTH | 2165 | HUMANS | 14801 |
| 5 | MHEALTH | 1381 | MALE | 14335 |
| 6 | MOBILE HEALTH | 612 | ADULT | 12436 |
| 7 | DIGITAL HEALTH | 585 | ARTICLE | 12337 |
| 8 | PANDEMIC | 532 | MIDDLE-AGED | 8117 |
| 9 | MENTAL HEALTH | 458 | AGED | 7006 |
| 10 | E-HEALTH | 448 | PANDEMIC | 5433 |

**Table S7.** Summary of studies analysing Google Trends data on telemedicine and other related topics.

| Reference | Location | Time Period | Main Findings |
| --- | --- | --- | --- |
| Hong et al [44] | USA | January-March 2020 | The study revealed a notable increase in US population-level interest in telehealth, correlating strongly with the rise in COVID-19 cases |
| Arshad et al [45] | World | January-July 2020 | A positive and fair correlation was established between global interest in telehealth and the reported new cases and deaths worldwide during the specified time frame |
| Jimenez et al [46] | Spain | February-May 2020 | The research indicated that the correlation between Google Trends search data and COVID-19 cases allowed the prediction of the pandemic's evolution up to 11 days in advance |
| Wong et al [47] | 50 most affected countries by COVID-19 | January-July 2020 | Across the 50 countries most affected by COVID-19, there was a general increase in interest and demand for telehealth services during the specified time period |
| Alonto et al [48] | World | 2016-2020 | Despite the global increase in the search of telehealth and telemedicine during the pandemic, teleneurology's interest remained stable, indicating a distinct pattern compared to the overall trend |
| Kinoshita et al [49] | Japan | March 2020 to October 2021 | The study highlighted significant differences in severe cases, deaths, and search volume of telehealth and COVID-19 between the first wave and subsequent periods. No long-term correlation was found between public interest in telehealth and COVID-19 |
| Van Kessel et al [50] | Canada, USA, UK, New Zealand, Australia, Ireland | February 2019 to August 2021 | Digital health search volumes immediately increased in all countries following the announcement of COVID-19 as a pandemic. While keyword variations existed, searches declined after the initial surge, sometimes returning to pre-pandemic levels |
| Leochico et al [51] | World | 2004-2020 | Searches for "telehealth," "telemedicine," and "telerehabilitation" peaked in March 2020, coinciding with lockdowns in many countries, especially in the Philippines. The term "telehealth" was more frequently searched in western countries, while "telemedicine" was relatively common in eastern countries |
